# Experience in Developing an FHIR Medical Data Management Platform to Provide Clinical Decision Support

**DOI:** 10.3390/ijerph17010073

**Published:** 2019-12-20

**Authors:** Ilia Semenov, Roman Osenev, Sergey Gerasimov, Georgy Kopanitsa, Dmitry Denisov, Yuriy Andreychuk

**Affiliations:** 1 Medlinx LLC, Saint-Petersburg 197101, Russia; ilia.semenov@medlinx.ru (I.S.); roman.osenev@medlinx.ru (R.O.); sergey.gerasimov@medlinx.ru (S.G.); dmitry.denisov@medlinx.ru (D.D.); yuriy.andreychuk@medlinx.ru (Y.A.); 2 National Center for Cognitive Research, ITMO University, Saint-Petersburg 197101, Russia

**Keywords:** semantic interoperability, FHIR, clinical decision support, integration

## Abstract

This paper is an extension of work originally presented to pHealth 2019—16th International Conference on Wearable, Micro and Nano Technologies for Personalized Health. To provide an efficient decision support, it is necessary to integrate clinical decision support systems (CDSSs) in information systems routinely operated by healthcare professionals, such as hospital information systems (HISs), or by patients deploying their personal health records (PHR). CDSSs should be able to use the semantics and the clinical context of the data imported from other systems and data repositories. A CDSS platform was developed as a set of separate microservices. In this context, we implemented the core components of a CDSS platform, namely its communication services and logical inference components. A fast healthcare interoperability resources (FHIR)-based CDSS platform addresses the ease of access to clinical decision support services by providing standard-based interfaces and workflows. This type of CDSS may be able to improve the quality of care for doctors who are using HIS without CDSS features. The HL7 FHIR interoperability standards provide a platform usable by all HISs that are FHIR enabled. The platform has been implemented and is now productive, with a rule-based engine processing around 50,000 transactions a day with more than 400 decision support models and a Bayes Engine processing around 2000 transactions a day with 128 Bayesian diagnostics models.

## 1. Introduction

This paper is an extension of work originally presented to pHealth 2019—16th International Conference on Wearable, Micro & Nano technologies for Personalized Health [1].

Decision support systems (DSSs) are currently being implemented to solve a high variety of clinical and environmental tasks. Successful implementation of a decision support system requires efficient planning strategies, a common understanding of decisions support goals, performance, and usability. This can increase acceptance and make the overall project successful [2]. The tasks that can be efficiently solved by decision support systems vary from the implementation of urban climate action plans [3] and support of road planning to the diagnosis of rare diseases [4].

Health care industry is increasingly becoming a knowledge-based community, connecting different providers, decreasing administrative costs, and improving quality and continuity of care. This creates challenges and opportunities for clinical decision support systems (CDSSs) that facilitate health care procedures in knowledge-based settings [5]. A CDSS is any computer program designed to help make clinical decisions [6,7]. This definition demonstrates the variety and evolution of clinical decision support from small, focused applications to large-scale platforms capable of storing and managing medical data to assist doctors and patients by delivering recommendations [8,9].

To provide efficient decision support, it is necessary to integrate CDSSs in information systems routinely operated by healthcare professionals, such as hospital information systems (HIS), or by patients deploying their personal health records (PHRs) [10]. CDSSs should be able to use the semantics and the clinical context of the data that were imported from other systems and data repositories [11,12,13]. 

Semantic interoperability becomes a key issue when it comes to communication between heterogeneous information systems [14]. One of the ways to connect different information systems is to build a platform that processes standard-based medical data and provides unified interfaces. Using clinical data exchange standards such as openEHR [15], CEN/ISO EN13606 [16,17,18], HL7 CDA [19], and fast healthcare interoperability resources (FHIR) [14] can provide data-level interoperability. These standards specify common electronic health record (EHR) data structures [20] and are widely used in clinical decision support systems [21,22]. One of the latest formats of EHR data specifications is the FHIR standard that provides data elements (“resources”) and an application programming interface (API) to retrieve and exchange electronic health records [23]. 

The current medical software ecosystem can be characterized by its high innovation needs, disturbing interactions between systems and hampering semantic interoperability [24]. To avoid such problems, developers can cluster software applications into small, easily supportable functional units that can be changed on demand without affecting other pieces of software. This approach is commonly referred to as microservice architecture [25].

Specifying standard interfaces, CDS Hooks (https://cds-hooks.org), provides a hook-based pattern for automatically invoking CDSS functions within routine clinical workflows [26]. This specification natively supports HL7 FHIR R4 to simplify the data flow, enabling easy integration of HISs and CDSS services. 

The experience shows that most of the CDSSs are standalone implementations focused on one clinical condition or workflow [27,28,29]. However, the implementation of sophisticated clinical decision support platforms that are capable of providing a full spectrum of clinical decision support functionality to various medical information systems is still missing. There is a persistent need for high-quality, effective platforms that will unify design, development, presentation, implementation, evaluation, and maintenance of all types of clinical decision support capabilities for clinicians, patients [30], and other stakeholders [31]. We advance existing experiences in implementing CDS platforms [32] by adding standard-based data interfaces and structures to deliver decision support features to different health care ecosystems. 

The goal of this research is to develop an FHIR-based microservice platform that integrates HISs and CDSSs into a unified information space.

## 2. Methods

### 2.1. Platform Architecture

Structurally, a CDSS platform was developed as a set of separate services, that is, nodes distributed in groups. Microservices communicate with each other asynchronously using the REST communication protocol.

### 2.2. Services

All the services operate through public contracts represented in FHIR JSON format, providing the unique resource identifier (URI) of the resources. Services utilize two interaction models: remote procedure call (RPC) and event-based interaction. Logs are sent to the central log service with a unique transaction ID.

**Figure 1:**
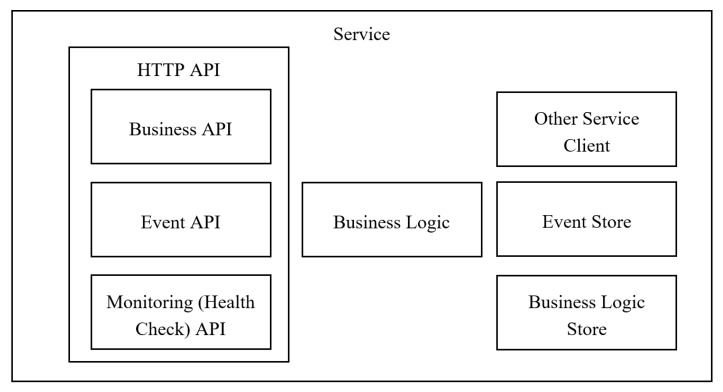
General model of service of the platform. API: application programming interface.

A model of the services is shown in Figure 1. Business API endpoints allow sending service-specific requests, for example, to return specific artifacts or terminology from the knowledge base. 

The Event API endpoint (e.g., HTTP/event) allows an external service to send event requests. So the service knows the status of other services of the platform.

The Health Check API endpoint (e.g., HTTP/health) returns the health status of the service to its handler to provide continual monitoring. The API endpoint handler performs various checks, such as

- the status of the connections to the infrastructure services used by the service instance;
- the status of the host, for example, disk space;
- application-specific logic.

Business Logic is the main part of the service that is responsible for the implementation of the feature the service is designed for, for example, the logic of loading facts from the database into an inference engine.

Event Store and Business Logic Store are responsible for managing and saving data related to the corresponding process of the service.

Other Service Client is responsible for active communication with other services of the platform, for example, sending the events and the results of the work.

Each service has an independent release cycle, so the public interfaces support versioning to provide consistent operation of the system.

### 2.3. Clinical Modelling

The CDSS platform requires designing a set of FHIR profiles suitable for the decision support. We used Forge (https://fhir.furore.com/forge/), the official HL7^®^FHIR^®^ profile editor, a desktop application for profile modelling and validation. We used Logical Observation Identifiers Names and Codes (LOINC) [33] and Systematized Nomenclature of Medicine—Clinical Terms (SNOMED CT) [34] codes to define the semantics of the medical concepts.

A “Patient” was used as the main resource of the platform. To provide semantic interoperability, the platform supports the following FHIR R4 [35] resources as input and output data:

1. CarePlan
2. MedicationRequest
3. ActivityDefinition
4. DetectedIssue
5. RiskAssessment
6. Questionnaire
7. QuestionnaireResponse
8. ResearchDefinition
9. PlanDefinition
10. Goal
11. Observation
12. Condition
13. FamilyMemberHistory
14. DiagnosticReport
15. Group
16. RequestGroup
17. AllergyIntolerance
18. Immunization
19. Procedure
20. Encounter
21. Appointment

### 2.4. Rule Engine

We accomplished extraction and conceptualization of the inference rules by manual formalization of clinical guidelines. We identified three types of inference rules: the definition, operation rule, and aggregation rule:

**Definition** is an atomic domain concept consisting of a nomenclature and a code.

**Operation Rule (OP)** is an operational inference rule that deals with a Definition that determines reference intervals for a Definition. For example,

- Definition_1. Value >1. Definition_2. Value = 0
- Operation Rule has the following structure:
  ○ Definition ID
  ○ Nomenclature
  ○ Code
  ○ Name
  ○ Operation
  ○ Value

**AggregateRule** is an Aggregation Rule (AG) that performs logical operations on Operation Rules. Aggregation Rules are built upon the IF-THEN paradigm, with the conditional combination of criteria (OPs) in the IF part and the suggested Artifacts in the THEN part. For example: if (Op1 and op2) or (op3 and op4) then Artifact.

An **Artifact** is a piece of a free-text recommendation that shall be included in the report as the result of the decision support. The system supports the following types of artifacts:

- **Scale** is used for the results of evaluation scales, questionnaires (as a coded value of the result or as a number);
- **Risk** is used to determine if there is a risk of any disease or condition;
- **Diagnosis** is used to evaluate the possible diagnosis based on laboratory or instrumental studies;
- **DiagnosticReport** is used for coded logic groups to interpret clinical observations;
- **ReferralRequest** is used to recommend specialist appointment and routing of patients;
- **ProcedureRequest** is used to recommend instrumental examinations;
- **DiagnosticRequest** is used to recommend laboratory tests;
- **Description** is used to describe the conclusions drawn (e.g., an explanation of the identified risk level);
- **Recommendation** is used for free text recommendations;
- **BehaviorRecommendation** is used for coded lifestyle recommendations.

When a specific FHIR instance of a patient’s case reaches a decision support system, patient data is first checked to match the formal definition of FHIR. Then, the data instances are registered in the fact database. Later, they are analyzed for the existence of a concept of a Definition object for every fact. The concepts that have corresponding Definitions are used for logical inference, where engine services create an inference sequence to generate a JSON file with artifacts to be included in the report. The resulting service creates a human-readable document based on the inference.

Depending on the input data, the Rule Engine can be used for different purposes:

- Calculation of risks of disease;
- Interpretation and monitoring of clinical observations;
- Analysis of medical services provided to a patient for compliance with the standards of the insurance company;
- Treatment plan management;
- Analysis of prescriptions for drug interactions and contraindications for prescribing.

**Figure 2:**
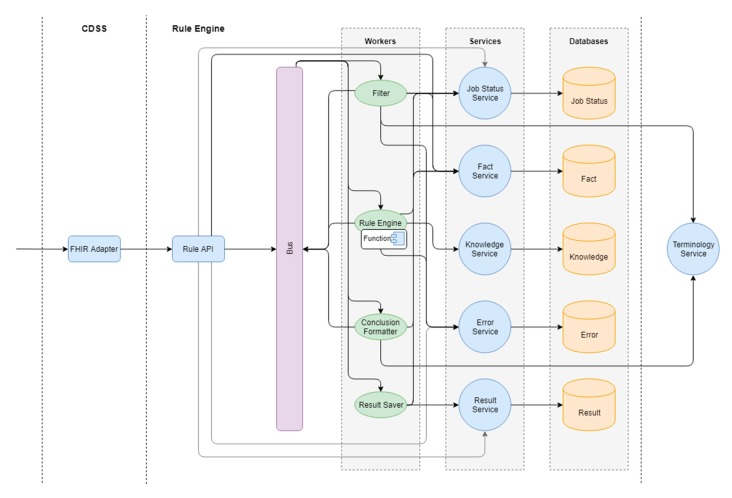
Services of the Rule Engine. FHIR: fast healthcare interoperability resources.

The Rule Engine includes the services presented in Figure 2.

- FHIR Adapter: the service converts data from the FHIR format into the internal Rule Engine format. This service also provides the possibility of Rule Engine invocation according to the CDS Hooks specification.
- Rule API: the service performs internal routing and saves processed data for further analysis.
- Filter: the service is responsible for filtering the data to the actual state, applicable to the mechanism of logical output.
- Rule Engine: the service is responsible for the logical inference mechanism based on the rules.
- Formatter: the service is responsible for formatting the results of logical inference.
- Api.KnowledgeService: the knowledge service is responsible for CRUD operations with the graphical knowledge base. Is used by the Rule Engine to search for rules, artifacts, and definitions.
- Api.FactService: fact service is responsible for preserving and providing facts at all stages of inference.
- Api.JobStatusService: the status service is responsible for creating new tasks and saving statuses.
- Api.ErrorService: the error service is responsible for saving and reporting errors that occur during the inference process.
- Api.ResultService: the result service is responsible for saving and providing the results obtained by the Rule Engine inference.
- TerminologyService is responsible for the storage and provision of medical terminology.

**Figure 3:**
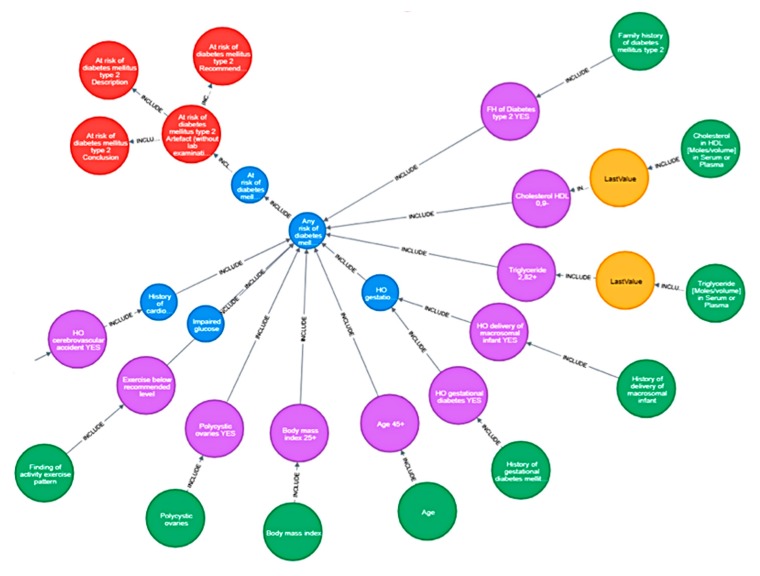
Example of a graphical model of rules for assessing the risk of type 2 diabetes.

The Rule Engine is based on production rules that are logical expressions represented in the form of a graph. An example of a graph model of rules for assessing the risk of type 2 diabetes is shown in Figure 3.

#### Rule Editor

**Figure 4:**
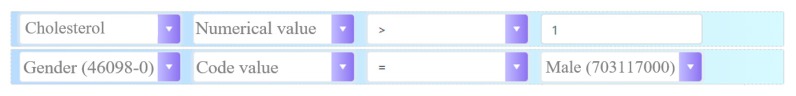
CDSS (clinical decision support system) rules editor.

**Figure 5:**
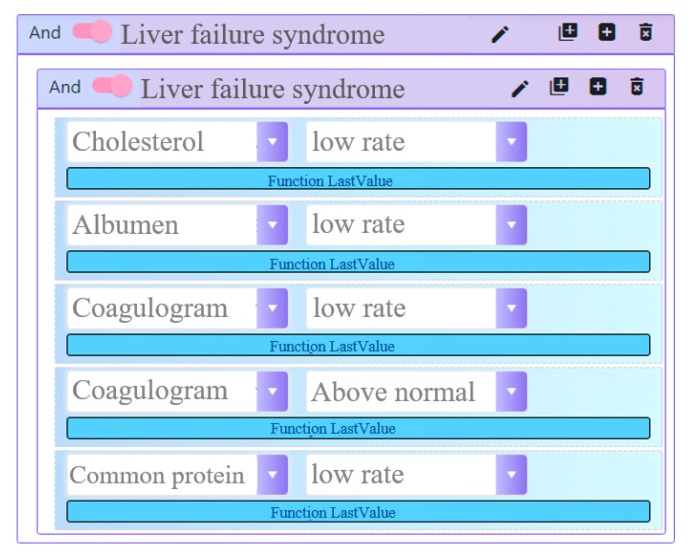
Rules combination.

**Figure 6:**
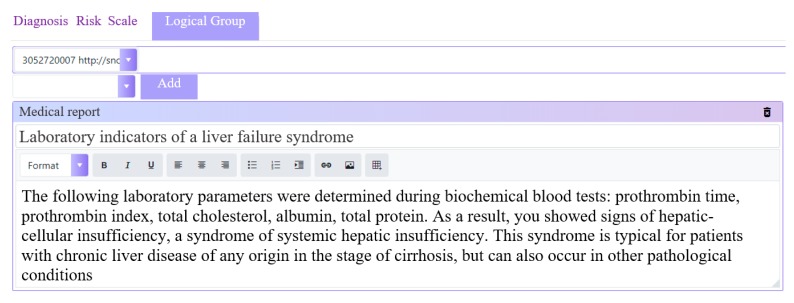
Free-text recommendations.

The rule editor has an intuitive interface that allows creating logical expressions (Figure 4, Figure 5 and Figure 6).

Conditions in logical expressions include comparison of actual values with the references and absolute values (Figure 4).

Conditions are combined into rules by logical operations AND, OR, NOT (Figure 5).

Conclusions of the rules are provided as Artifact type and contain additional information about the interpretation of laboratory analyses, recommendations for appointment, recommendations for additional instrumental and laboratory studies, and so forth. (Figure 6).

### 2.5. Bayes Engine

A Bayes Engine is a DSS system based on Bayesian Logic. Bayesian Statistics allow a system to make conclusions about the presence of pathology in the patient in the conditions of high uncertainty. Bayes Engine models enable to carry out initial patient interview, guide the patient to the right doctor, and to assist the doctor in making a preliminary diagnosis. Models are developed by expert physicians on the basis of generally accepted clinical guidelines and scientific publications with a high level of evidence.

Bayes Engine services were implemented using

1. C# + .net core 2.2 for the core logic;
2. asp.net core 2.2—for the web interface;
3. Infer.NET (ML.net)—a framework for running Bayesian inference in graphical models. In our case it was used for probabilistic programming.
4. PostgreSQL 11 for data storage.

**Figure 7:**
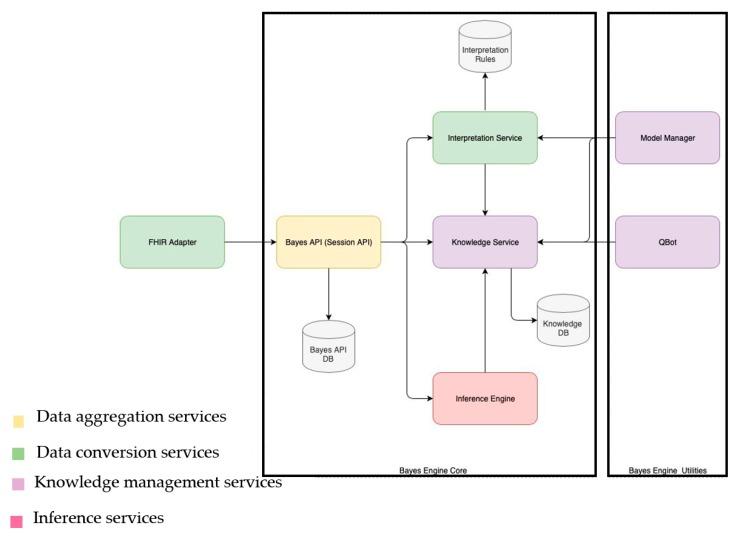
Bayes Engine component diagram

Bayes Engine includes the following services (Figure 7):

- FHIR Adapter: the service converts data from the FHIR format into the internal Bayes Engine format. Furthermore, this service provides the possibility of Rule Engine invocation according to the CDS Hooks specification.
- Bayes API: the service performs internal routing and stores processed data for further analysis;
- Interpretation Service: the service performs data processing before calculation on Bayesian networks;
- Knowledge Service: the service stores Bayesian models created by experts and collects probabilities used for calculations;
- Inference Engine: the service performs inference on the basis of Bayesian models;
- Qbot: the service validates inference results and models with experts in the learning mode;
- Model Manager: supports model management in the Knowledge Service (CRUD on models).

The process of creating diagnostic Bayesian models includes the following steps:

1. Creating a model prototype;
2. Model structure validation by experts;
3. Improvement of the model structure;
4. Definition of probabilities;
5. Validation of the model by experts using real validated clinical cases.

The Bayesian network editor is used to create diagnostic models. A model is a graph with nodes representing diagnoses, risk factors, symptoms, objective signs, as well as laboratory and instrumental data. Edges between the nodes indicate cause–effect relationships and correlations. All nodes are coded using international classifiers (LOINC and SNOMED CT).

### 2.6. CDS Hooks

For the integration of the CDS Hooks API into the CDSS platform, we utilized the latest version of the specification of CDS Hooks (1.0 STU).

To ensure interoperability, the CDS Manager service is included in the CDSS platform to perform the following tasks:

1. Store a directory of available CDSS services on the platform, provide registration, and allow the client application to configure which CDSS to use for specific needs.
2. Provide proxying calls from the client to the selected CDSS and collect usage statistics.

**Figure 8:**
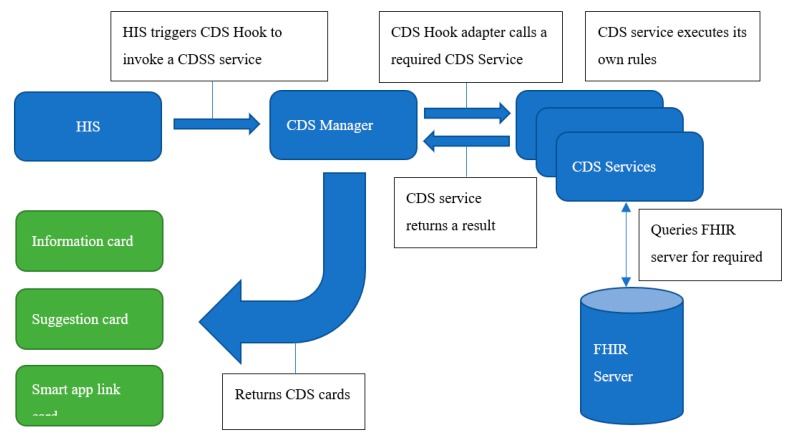
CDS Hook interaction workflow. HIS: hospital information system.

**Figure 9:**
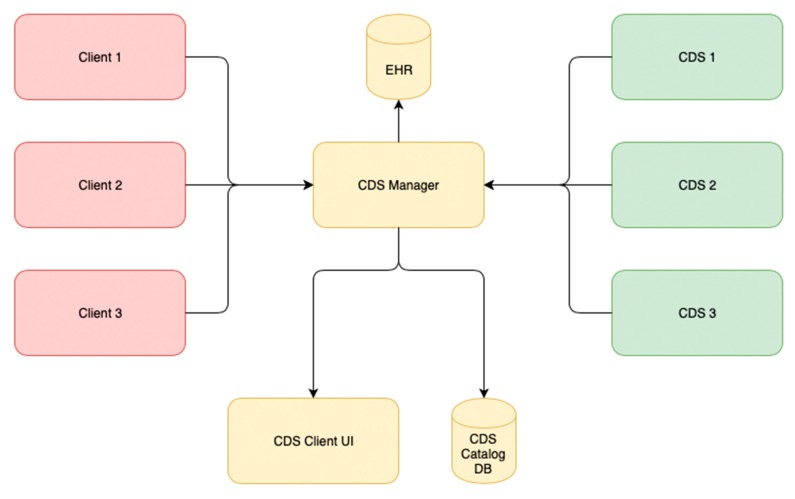
CDS manager structure model. EHR: electronic health record.

The process and components of interaction between HISs and the CDS manager are shown in Figure 8 and Figure 9. 

The CDS manager allows connections from any CDSS services that implement the CDS Hooks 1.0 specification.

To implement the CDS Hooks, we consequently implemented three main features:

1. RESTful interface based on the CDS Hooks specification. This included support of the defined format of the RESTful body and the return of the CDS results as cards;
2. A FHIR platform adaptor was adjusted to support individual FHIR connection for each EHR session;
3. Implementation of the data points required by the CDS Hooks API. This included the management of hooks and the data points that needed to be pre-fetched by the calling HIS.

## 3. Results

### 3.1. Rule Engine

A Rule Engine is an inference system based on production rules. It receives patient-related observations as an input and returns conclusions about the patient’s condition with recommendations for further actions. The rules that are incorporated into the system are referenced by clinical guidelines and scientific publications with a high level of evidence. 

In total, we have modelled 365 nodes of laboratory test components, 5084 nodes of inference rules, 49,932 nodes, and 3072 blocks of text for medical certificates.

We have developed interpretation algorithms for the following 11 groups of codes of the International Statistical Classification of Diseases and Related Health Problems (ICD-10):

N30 Cystitis

N04 Nephrotic syndrome

N10 Acute pyelonephritis

K75 Other inflammatory liver diseases

K71 Toxic liver disease

K81 Cholecystitis

K85 Acute pancreatitis

E05 Thyrotoxicosis

D50 Iron deficiency anemia

D72 Other disorders of white blood cells

N41 Inflammatory diseases of prostate.

### 3.2. Bayes Engine

**Figure 10:**
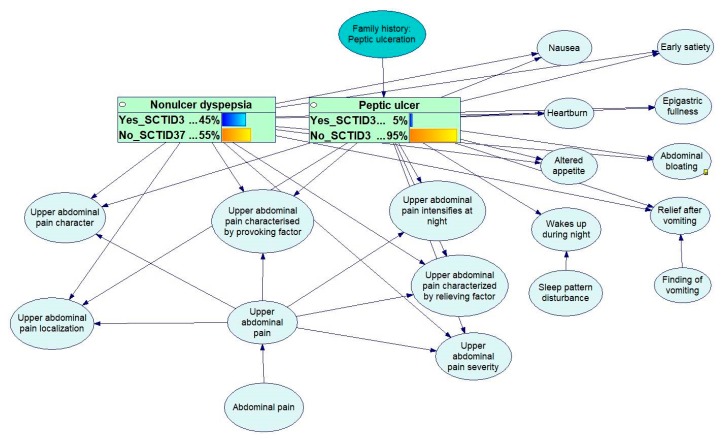
Example of the structure of a probabilistic model.

An example of model structure is presented in Figure 10.

**Figure 11:**
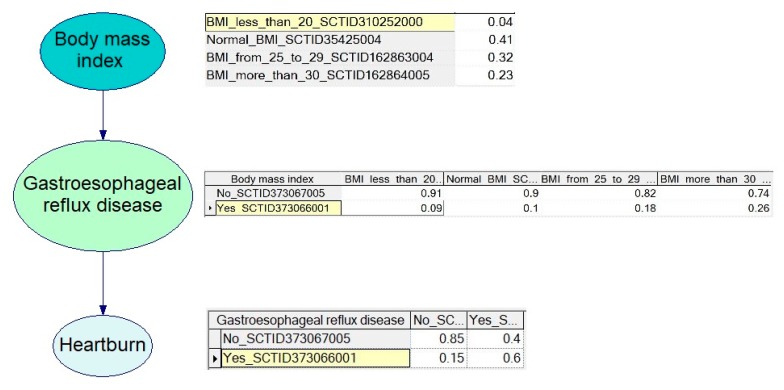
Example of probability tables for Gastroesophageal reflux disease.

For each node, experts specify disease-specific stratification. To determine the probability values, experts use statistical data from clinical guidelines and standards. An example of probability tables is shown in Figure 11. Each model is validated by at least two experts before it can be accepted for production. A model is accepted only after a consensus between two experts is reached. 

We have implemented Bayesian diagnostics models for 128 diagnoses.

#### Bayes Engine Workflow Example

**Figure 12:**
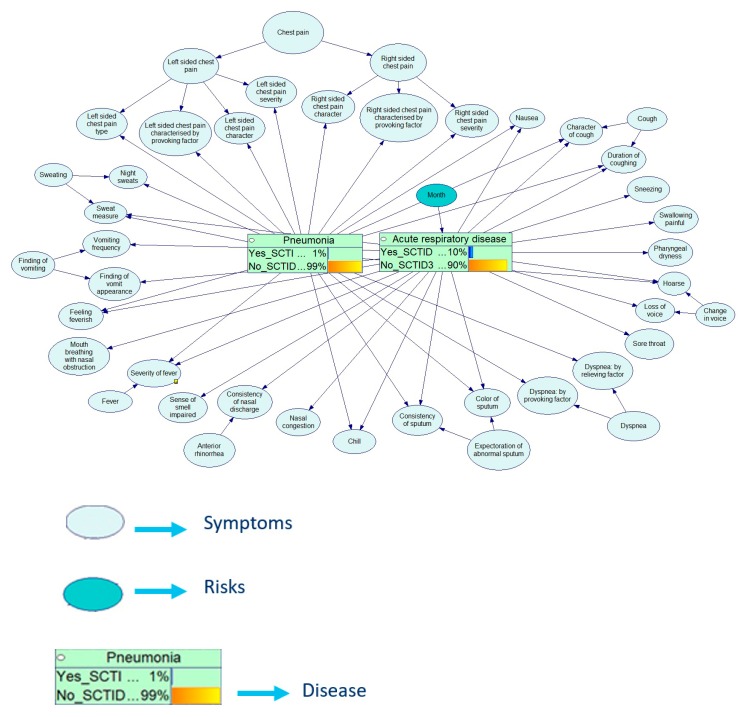
Initial state of a Bayesian network.

**Figure 13:**
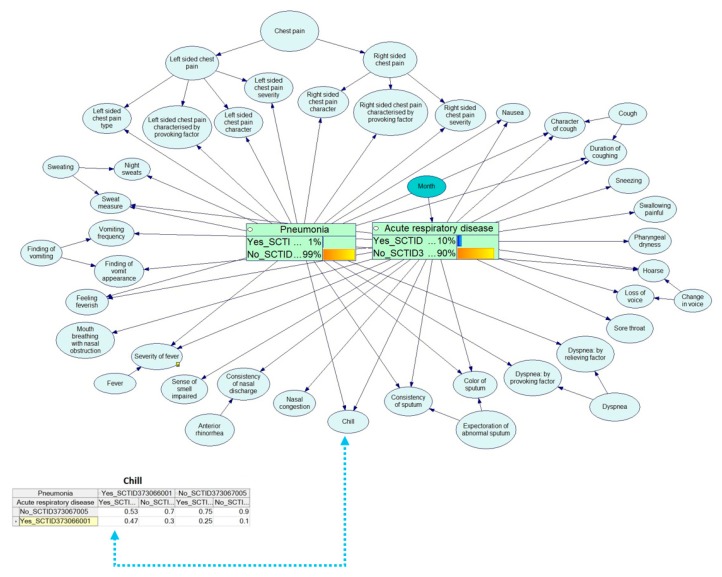
Probability table for a chill symptom.

**Figure 14:**
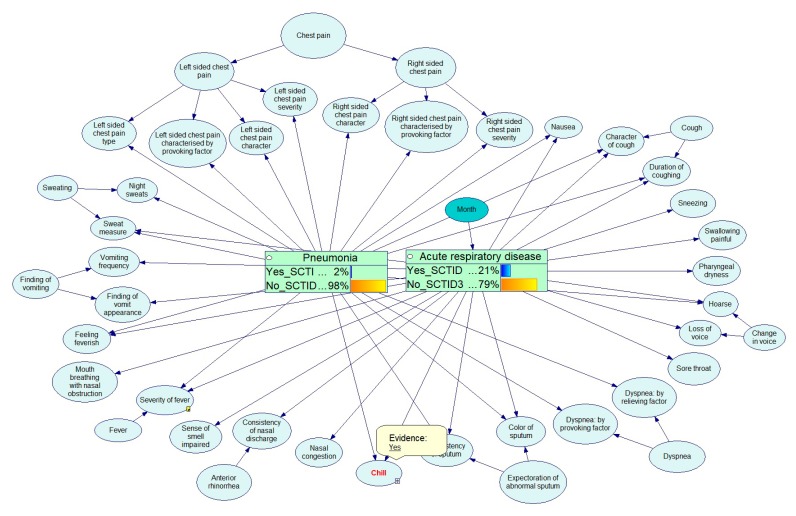
Service has received data on the presence of chill.

**Figure 15:**
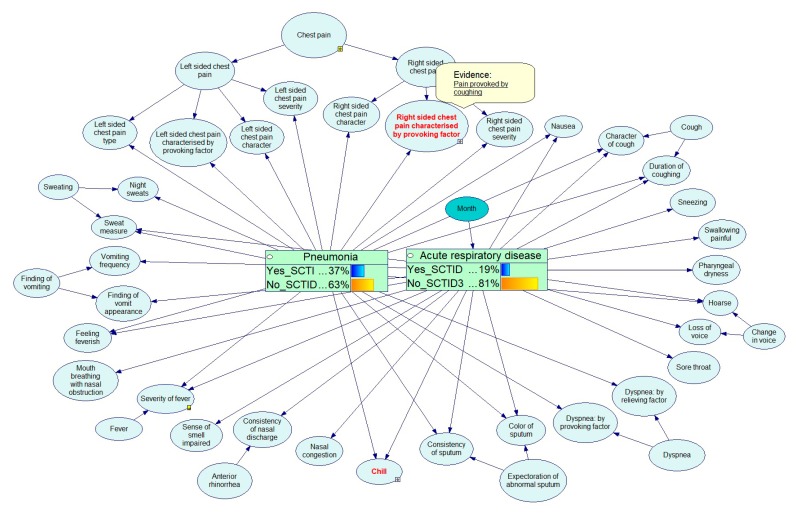
Service has received data that the patient has a chest pain on the right side during cough.

**Figure 16:**
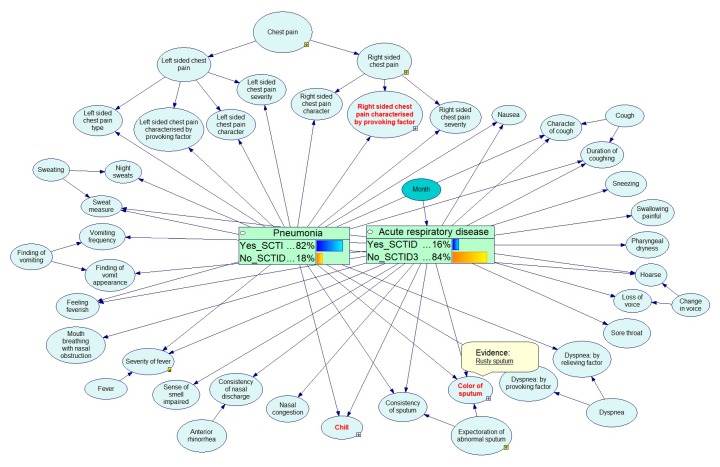
Service has received data that the patient has rusty sputum coming off.

The example below shows the Bayesian calculations for the diagnostics of respiratory diseases. Figure 12, Figure 13, Figure 14, Figure 15 and Figure 16 demonstrate the process of how the Bayesian engine calculates probabilities of pneumonia and acute respiratory disease after receiving new symptoms. The figures describe for each step the recalculation of probabilities of Pneumonia and Acute respiratory disease when introducing new facts: presence of chill, right-sided chest pain, and color of a sputum. 

## 4. Discussion

In this paper, we focused on the development of the core components of a CDSS platform, that is, its communication services and logical inference components. The most valuable characteristic of the proposed platform is the compatibility and interoperability of its services, facilitating the development of a transparent and pluggable CDSS system. CDSS systems can be implemented as standalone CDSS solutions or be integrated into a clinical workflow operated by an HIS. 

Our research advances the state of the art in semantically interoperable clinical decision support systems. The previously developed FHIR-based CDSSs [36,37,38] focused on one particular decision support task. Our platform allows definition of multiple purpose decision support models, provided that they (a) use FHIR resources as a data source and (b) are based on production rules or Bayesian Logic. In comparison with the CDSSs developed based on other standards for medical data exchange, such as openEHR [39], the proposed platform does not require modelling of specific data sets for each task and can process standard FHIR resources instead. 

### 4.1. Architecture

Each type of a decision support task in the platform has its own microservice to allow a continued delivery of new features and decision support models. All interaction between services is processed by a common API gateway, which supports versioning. This approach allows distinguishing responsibilities between services and performing parallel development of the services by different dedicated teams. 

### 4.2. Implications and Future Use

The results of the presented research have a valuable impact on design and implementation of large-scale clinical decision support systems. It is very important to make clinical decision support systems valid and easily acceptable by users. To accomplish this, we recommend to run a pilot application of the decision support system by the experts to verify and validate the rules to increase the system’s reliability and acceptance.

A FHIR-based CDSS platform addresses the ease of access to the clinical decision support services by providing standard-based interfaces and workflows. This type of CDSS may be able to improve the quality of care for doctors who use HISs without CDSS features. The HL7 FHIR interoperability standards make this platform usable by all HISs that are capable of integrating with the HL7 FHIR standard.

### 4.3. Operation

At the moment, the platform processes about 15,000 orders a day with foreseen 50,000 orders a day. The system is able store up to 50 million orders, with the average of five tests in each one to provide the capacity for the next 7 years of operation.

During the operation of the platform, we discovered the similar weak points of the developed micro-service-based system as reported by other micro-service-based decision support systems [40,41,42]. Operation complexity has increased due to the introduction of service discovery, service backup, and automatic restore. Maintenance also became complicated due to complex logging systems, where each service produces its own logs. We observed a relatively moderate, but nevertheless observable slowdown of transaction handling due to the distributed processing.

## 5. Conclusions

In this paper, we proposed a cloud-based interoperable multipurpose CDSS platform system that makes use of semantic technologies based on the HL7 FHIR standard. We implemented two types of knowledge modeling formalisms in the platform, namely production rules and Bayesian Logic. A flexible knowledge editor supports clinical experts implementing decision support models for any purpose based on the provided formalisms. The platform is capable of provision of decision support services for any FHIR-enabled HIS with standard CDS Hooks interfaces. It makes access to CDS services easier and promotes their use in routine clinical professes to improve efficiency and efficacy of health care. The platform was implemented and is now productive, processing around 50,000 transactions a day with more than 400 decision support models.
